# Muscarinic Receptor Signaling in Colon Cancer

**DOI:** 10.3390/cancers3010971

**Published:** 2011-03-02

**Authors:** Erik C. von Rosenvinge, Jean-Pierre Raufman

**Affiliations:** 1 University of Maryland School of Medicine, Division of Gastroenterology & Hepatology, 22 S. Greene Street, N3W62, Baltimore, MD 21201, USA; E-Mail: jraufman@medicine.umaryland.edu; 2 Department of Veterans Affairs, VA Maryland Health Care System, 10 North Greene Street, Baltimore, MD 21201, USA

**Keywords:** colon cancer, bile acids, muscarinic receptors, epidermal growth factor receptors

## Abstract

According to the adenoma-carcinoma sequence, colon cancer results from accumulating somatic gene mutations; environmental growth factors accelerate and augment this process. For example, diets rich in meat and fat increase fecal bile acids and colon cancer risk. In rodent cancer models, increased fecal bile acids promote colon dysplasia. Conversely, in rodents and in persons with inflammatory bowel disease, low-dose ursodeoxycholic acid treatment alters fecal bile acid composition and attenuates colon neoplasia. In the course of elucidating the mechanism underlying these actions, we discovered that bile acids interact functionally with intestinal muscarinic receptors. The present communication reviews muscarinic receptor expression in normal and neoplastic colon epithelium, the role of autocrine signaling following synthesis and release of acetylcholine from colon cancer cells, post-muscarinic receptor signaling including the role of transactivation of epidermal growth factor receptors and activation of the ERK and PI3K/AKT signaling pathways, the structural biology and metabolism of bile acids and evidence for functional interaction of bile acids with muscarinic receptors on human colon cancer cells. In murine colon cancer models, deficiency of subtype 3 muscarinic receptors attenuates intestinal neoplasia; a proof-of-concept supporting muscarinic receptor signaling as a therapeutic target for colon cancer.

## 1. Introduction

Each year, ∼150,000 people in the United States are diagnosed with colon cancer (http://www.cancer.org/), the leading gastrointestinal (GI) cause of death [1]. Early colon cancer, limited to the colon mucosa, is readily treated by surgery or endoscopy with an excellent outcome, greater than 95% five-year survival. However, more invasive and metastatic colon cancers respond poorly to current therapies; consequently, one-third of patients with colon cancer die from the disease. Novel chemotherapeutic agents and treatments that target the ligand-binding and kinase domains of epidermal growth factor receptors have provided limited improvement in survival. Activating mutations in key genes that code for effector molecules downstream of epidermal growth factor receptors (EGFR) (e.g., BRAF and KRAS) are frequently responsible for resistance to chemo- and radiation therapy [2]. Hence, it is important to identify novel targets for colon cancer prevention and treatment.

Colon cancer arises as a consequence of the adenoma-carcinoma sequence which is associated with accrual of multiple somatic gene mutations [2]; environmental factors accelerate and augment this process. For example, diets rich in meat and fat increase fecal bile acids and colon cancer risk [3]. In rodent cancer models, increased fecal bile acids promote colon dysplasia [4]. Conversely, in rodents [5,6] and persons with inflammatory bowel disease [7], low-dose ursodeoxycholic acid treatment alters fecal bile acid composition and attenuates colon neoplasia. Hence, fecal bile acids appear to play an important role in the promotion of colon cancer. A serendipitous observation [8] led us to hypothesize that bile acids interact functionally with intestinal muscarinic receptors [9]. Of five muscarinic receptor subtypes (designated CHRM1-5), those that activate phospholipid turnover (CHRM1,3,5) are conditional oncogenes when expressed in cells capable of proliferation [10]. CHRM3 are expressed widely in the GI tract and in colon cancer cells [11]; in one small study, compared to adjacent normal tissue, in 63% of colon cancer specimens CHRM3 expression was increased up to 8-fold [12]. Collectively, these observations identify elements of muscarinic receptor signaling as novel targets to prevent and treat colon neoplasia.

## 2. Muscarinic Receptors

**Figure 1.**
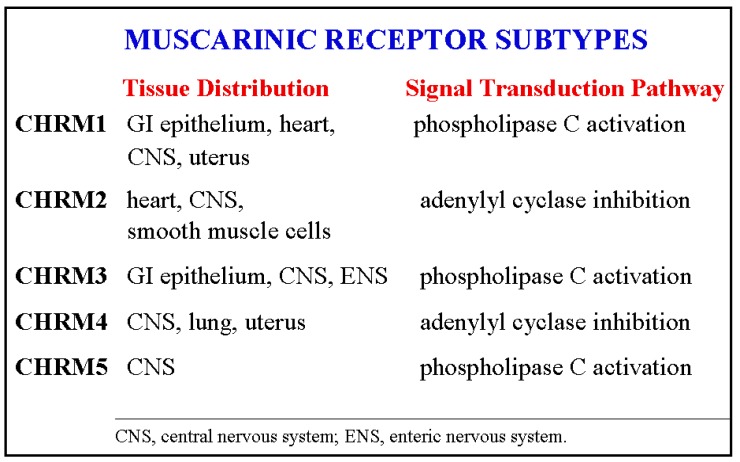
Tissue distribution and signal transduction pathways of the five muscarinic receptor subtypes.

Muscarinic receptors are members of the large family of G-protein coupled receptors (GPCR) that activate 5′-phosphate (G) proteins. G-proteins regulate a wide-range of biological processes by modulating the activity of adenylyl cyclase, phosphatidylinositol lipid turnover and ion channels [13,14]. Like other G-protein coupled receptors, muscarinic receptors have seven transmembrane helical domains connected by three extracellular and three intracellular loops. Five receptor subtypes, *CHRM1-5*, identified by molecular cloning techniques, modulate cell function by different post-receptor signaling pathways (Figure 1) [15]. Activation of CHRM1, CHRM3 and CHRM5 results in phospholipid turnover and changes in cell calcium concentration. Activation of CHRM2 and CHRM4 results in inhibition of adenylyl cyclase and reduced levels of cAMP.

Muscarinic receptors are long-established as instrumental in neuronal signaling [16]. In addition to the GI tract, muscarinic receptors are expressed normally throughout the body, including the brain [17], eye [18], heart [19], vasculature [20,21], lung [22], bladder [23] and uterus [24]. More recently, novel observations demonstrated muscarinic receptor expression and activation in various cancers including those arising in the brain, breast, colon, skin, lung and prostate [25].

In the GI tract primarily CRHM1, CRHM2 and CRHM3 are expressed. Gastric acid secretion by parietal cells is regulated by CHRM3 [26,27] and pepsinogen secretion from chief cells is regulated by a mixture of CHRM1 and CHRM3 [28-30]. In normal colonic epithelium both CHRM1 and CHRM3 are expressed [12,31], while in colon cancer upregulation of CHRM3 expression is suggested [12].

## 3. Muscarinic Receptor Ligands

In humans, acetylcholine (ACh) and conjugated secondary bile acids are the only known endogenous muscarinic receptor ligands. Production of ACh by neurons and its neurotransmitter properties are well-characterized [32]. Recently, non-neuronal production of ACh and its importance in cancer has become an active area of investigation [25]. In addition to signaling via muscarinic receptors, as detailed in a recent review non-neuronal ACh interactions with nicotinic receptors may promote cancer development [33]. Following an unanticipated observation [8], our laboratory demonstrated that secondary bile acids functionally interact with muscarinic receptors expressed in Chinese hamster ovary and colon cancer cells [34-36].

### 3.1. Acetylcholine

First discovered as naturally occurring by Sir Henry Dale and his colleague Arthur Erwins in 1913 and subsequently characterized as a neurotransmitter by Otto Loewi in the 1920s [37], ACh is the principal muscarinic receptor ligand. Neuronal production of ACh is catalyzed principally by the enzyme cytoplasmic choline acetyltransferase (ChAT) [32]. Due to the efficiency of acetylcholinesterases (AChE) at synaptic junctions, neuronal ACh is quickly hydrolyzed into choline, acetate and water, and, therefore, its effects are likely limited to synaptic transmission. However, the presence of ChAT and production of ACh by non-neuronal tissue was first reported in the 1930s [38]. Subsequently, ChAT expression has been reported in multiple cell types [25]. An additional mechanism of ACh production utilizes the widely-expressed enzyme carnitine acyltransferase (CrAT).

Using quantitative real-time PCR, our group demonstrated that H508, WiDr, and Caco-2 human colon cancer cell lines all express ChAT, that H508 and Caco-2 cells release ACh into cell culture media, and that muscarinic receptor antagonists inhibit proliferation of unstimulated H508 cells by about 40% [39]. Immunohistochemical staining of surgical specimens revealed that normal colonic epithelium has weak or no staining for ChAT, whereas half of the colon cancer specimens examined exhibited moderate to strong staining [39]. Collectively, these findings provide strong evidence that ACh is an autocrine growth factor in colon cancer.

### 3.2. Bile Acids

**Figure 2.**
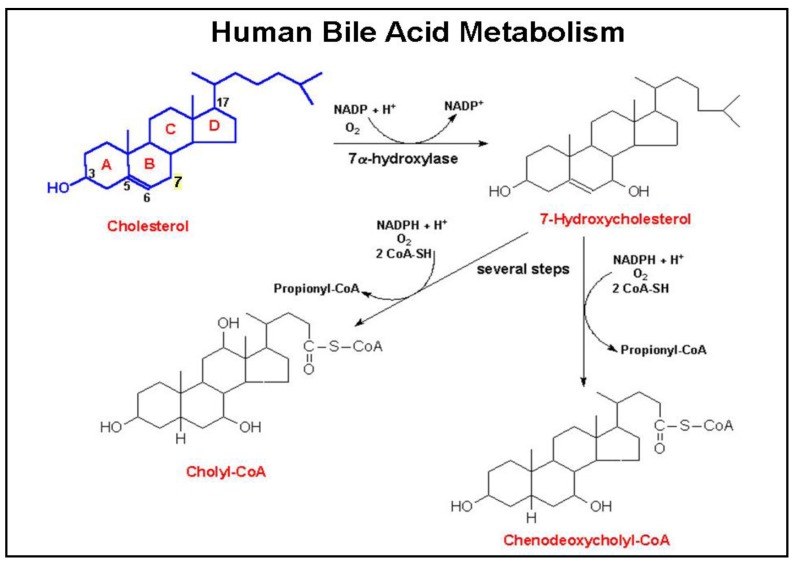
Human bile acid metabolism. Human bile acids derive primarily from cholesterol metabolism in the liver. The 7α-hydroxylase enzyme is the rate-limiting step in the formation of 7-hydroxycholesterol, the precursor of cholic and chenodeoxycholic acid.

Bile acids are produced by hepatocytes and secreted into bile, predominantly after conjugation (amidation) with taurine or glycine [40]. As illustrated in Figure 2, primary human bile acids derive from cholesterol metabolism following a series of steroid nucleus hydroxylations. These primary bile acids, cholic and chenodeoxycholic acids, are secreted into the small intestine where bacterial dehydroxylases, primarily from Clostridia species, modify these molecules to form the secondary bile acids, deoxycholic and lithocholic acids, respectively. Both primary and secondary bile acids are absorbed in the distal ileum by a highly efficient transporter on the apical surface of enterocytes (apical sodium-dependent bile acid transporter, commonly referred to as ASBT). Via the portal circulation, bile acids are transported back to the liver where secondary bile acids undergo conjugation (amidation) with either taurine or glycine, and are secreted into the biliary tree and intestines. Reduced expression or inactivation (mutation) of ASBT results in reduced bile acid up-take from the distal small intestine and increased fecal bile acids. The bile acid nuclear receptor Farnesoid X Receptor (FXR), a key regulator of bile acid homeostasis, is expressed widely in the GI tract. The actions of FXR and its possible role in colon cancer have recently been reviewed [41].

Novel observations in our lab revealed the interaction of bile acids with muscarinic receptors. In the course of examining actions of bile acids on pepsinogen secretion by gastric chief cells we found that taurine conjugates of lithocholic acid bind to muscarinic receptors, increase inositol phosphates (IP), and stimulate secretion by a cholinergic mechanism [8]. In collaboration with the Frucht lab, we revealed the functional interaction of lithocholic acid conjugates with CHRM3 receptors on a human colon cancer cell line [34]. Specifically, in H508 colon cancer cells which express CHRM3, lithocholytaurine (LCT) was shown to stimulate dose-dependent increases in cell proliferation up to 200% compared to control, dose-dependent inhibition of radioligand binding, and increased IP formation. No such changes were observed in a colon cancer cell line (SNU-C4) that does not express CHRM3 [34]. Molecular modeling suggests that the functional interaction of bile acids with muscarinic receptors is due to shape and surface charge similarities to ACh [36].

## 4. Muscarinic Receptor Signaling in Colon Cancer

**Figure 3.**
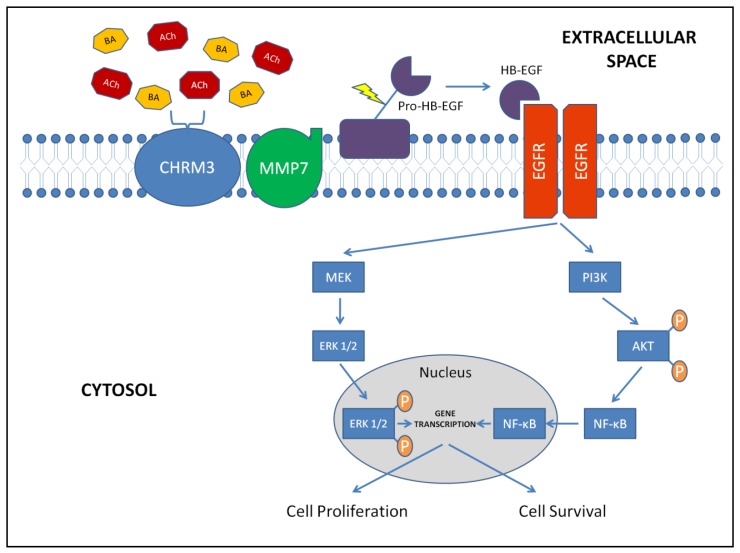
Muscarinic signaling in colonic epithelial cells. Acetylcholine (ACh) and secondary bile acids (BA) activate extracellular muscarinic receptors (CHRM3). Activated CHRM3 stimulates matrix metalloproteinase-7 (MMP7) to cleave heparin binding epidermal growth factor (HB-EGF) from Pro-HB-EGF. HB-EGF transactivates epidermal growth factor receptors (EGFR) resulting in intracellular signaling via both the MEK/ERK and PI3K/AKT signaling pathways. Phosphorylation of ERK and AKT are shown (P) and promote translocation of ERK and NF-κB from the cytosol into the nucleus. Resultant gene transcription promotes cell proliferation and cell survival (inhibition of apoptosis), both hallmarks of neoplasia.

*In vitro* studies of human colon cancer cell lines revealed the principal pathways of muscarinic receptor signaling in colon cancer (Figure 3). More recently, *in vivo* murine models of colonic neoplasia confirmed the importance of muscarinic signaling in colon cancer.

### 4.1. In Vitro Studies of Muscarinic Receptor Signaling in Colon Cancer

Using H508 human colon cancer cells with high-level expression of CHRM3 [42] we identified bile acids as CHRM3 agonists and revealed that, as with ACh [43], proliferative actions of bile acids require cross-talk between CHRM3 and epidermal growth factor receptors (EGFR) and post-receptor ERK1/2 activation [44,45]. Bile acids stimulated proliferation of H508 cells that co-express CHRM3 and EGFR, but did not alter proliferation of SNU-C4 cells that express EGFR but not CHRM3 [44]. The requirement for CHRM3 activation was confirmed using chemical inhibitors of muscarinic receptor activation [44,45]. Transactivation of EGFR by GPCR (e.g., CHRM3) is a common feature of mitogenic signaling [46]. Work detailed in [44] reveals that: (1) In H508 cells that co-express CHRM3 and EGFR, bile acids stimulate cell proliferation. (2) Bile acids do not alter proliferation of SNU-C4 cells that express EGFR but not CHRM3. (3) Proliferative actions of bile acids are inhibited by CHRM3 antagonists. (4) Rapid and reversible post-EGFR ERK activation is detected with micromolar levels of bile acids that stimulate cell proliferation [45] and were detected by us in the human cecum [47]. Collectively, these findings define efficacious bile acid concentrations, emphasize the importance of co-expression of CHRM3 and EGFR for bile acid-induced colon cancer cell proliferation, and identify the key role of post-EGFR ERK signaling.

EGFR actions on cell proliferation and survival are mediated by several signaling cascades. Bile acids protect colon cancer cells from apoptosis by EGFR-, PI3K/AKT-dependent mechanisms that involve activation of NF-κB [48]. Treatment with bile acids increases resistance of colon cancer cells to TNF-α- and UV-induced apoptosis, and stimulates nuclear translocation and transcriptional activity of NF-κB [49]. Reduced activation of NF-κB using an IκBα super-repressor (AdIκBSR) or chemical inhibitors attenuates anti-apoptotic actions of bile acids [49]. Bile acid-induced NF-κB activation and rescue from apoptosis is regulated by PI3K/AKT signaling downstream of EGFR [49]. Bile acid-induced resistance to TNF-α- and UV-stimulated apoptosis requires activation of AKT; both NF-κB activation and anti-apoptotic actions of bile acids were attenuated when AKT expression and activation were reduced by transfection with mutant *AKT* or treatment with an AKT inhibitor, respectively [49]. These observations in H508 cells were confirmed in another human colon cancer cell line (HT-29 cells) [49]. Collectively, these findings demonstrate that, downstream of EGFR activation, bile acid-induced PI3K/AKT and NF-κB activation regulate colon cancer cell apoptosis and survival. These actions of bile acids are likely to be important for colon cancer cell resistance to chemo- and radiation-therapy.

Matrix metalloproteinases (MMPs) catalyze EGFR ligand release. Work detailed in [45] reveals that MMP7 mediates bile acid-induced activation of EGFR by catalyzing release of HBEGF (Figure 3). Bile acid-induced H508 cell proliferation is blocked by anti-HBEGF antibody, by CRM197, an agent that blocks HBEGF release, and by GM6001, a non-selective MMP inhibitor [45]. Recombinant MMP7 and HBEGF both mimic proliferative actions of bile acids. Using q-PCR, immunohistochemistry and ELISA, we showed that H508 colon cancer cells express MMP7 and that *MMP7* gene transcription is induced by bile acids. The half-life of *MMP7* mRNA (∼16 h) was not altered indicating that *MMP7* expression is stimulated at the transcriptional level [50]. Proliferative actions of bile acids are blocked by anti-MMP7 antibody and knockdown with MMP7 siRNA [45]. Collectively, these findings identify molecular mechanisms leading from bile acid interaction with CHRM3 to activation of EGFR (Figure 3). The role of muscarinic receptor ligand induction of MMP1 and 10 gene transcription remains to be determined.

### 4.2. In Vivo Studies of Muscarinic Receptor Signaling in Colon Cancer

To investigate the importance of CHRM3 expression *in vivo* we utilized the azoxymethane (AOM) model of colonic neoplasia in wild type (WT) and Chrm3-deficient (*Chrm3*^−/−^) mice [31]. When compared to AOM-treated WT mice, AOM-treated *Chrm3*^−/−^ mice revealed 40% reduction in colon tumor number and 60% reduction in tumor volume. Additionally, cell proliferation (determined by BrdU staining) was reduced by 43% in *Chrm3*^−/−^ compared to WT mice. These findings confirmed the importance of CHRM3 receptor expression in colon neoplasia. To determine the therapeutic potential of muscarinic antagonists in human colon cancer, additional studies, including investigations of specific CHRM3 antagonists and human trials, are required.

## 5. Therapeutic Targets of Muscarinic Receptor Signaling in Colon Cancer

The complexity of the muscarinic signaling cascade in colon cancer (Figure 3) provides many potential targets for therapeutic intervention. Reducing muscarinic receptor ligands by altering colon bile acid composition (e.g., administration of low-dose ursodeoxycholic acid) was shown to prevent colon cancer in patients with inflammatory bowel disease [7], and may have a role after onset of colonic malignancy. Agents that decrease ACh production (e.g., choline acetyltransferase inhibitors) could attenuate the pro-proliferative effects of muscarinic signaling, but unless delivered locally would likely have unacceptable toxicity given the importance of ACh in neuronal signaling. As described above, muscarinic receptor antagonists can decrease intestinal neoplasia in a murine model of colon cancer; the role for these agents in human colon cancer should be explored.

## 6. Conclusions

Colon epithelial cells express muscarinic receptors; this is increased in colon cancer cells. Principal muscarinic receptor ligands in colon cancer are both neuronal and non-neuronal ACh, and secondary bile acids. Once activated, muscarinic receptors transactivate EGFR via MMP-7-catalyzed cleavage of HB-EGF (an EGFR ligand) from pro-HB-EGF. Post-EGFR signaling is mediated by two intracellular cascades, the MEK/ERK and PI3K/AKT/NF-κB pathways. These pathways promote gene transcription favoring cell proliferation and survival, both hallmarks of neoplasia. Recent *in vivo* studies confirm the importance of muscarinic signaling in colon cancer, demonstrating the promise that inhibitors of muscarinic signaling may be a novel approach to treat this disease.
